# Pathologic complete response after laparoscopic surgery following treatment with nivolumab and ipilimumab for anticancer drug-resistant MSI-high descending colon cancer: a case report and literature review

**DOI:** 10.1186/s40792-022-01580-w

**Published:** 2022-12-27

**Authors:** Hiroshi Sawayama, Yuji Miyamoto, Katsuhiro Ogawa, Mayuko Ohuchi, Yuki Hisano, Moeko Kato, Hiroki Tubakihara, Naoya Yoshida, Hideo Baba

**Affiliations:** 1 Department of Gastroenterological Surgery, Graduate School of Medical Sciences, Kumamoto University, 1-1-1, Honjo, Chuo-Ku, Kumamoto, 860-8556 Japan; 2 Department of Surgery, Kumamoto General Hospital, 10-10, Toricho, Yatushiro, Kumamoto, 866-0856 Japan

**Keywords:** Nivolumab, Ipilimumab, Complete response, Laparoscopic resection

## Abstract

**Background:**

Preoperative treatment is performed for locally advanced colon cancer with extensive tumor proximity or suspected invasion of skeletal muscles, major organs, and blood vessels. Oxaliplatin-based regimens are often used in preoperative chemotherapy. However, microsatellite instability (MSI)-high colorectal cancer is often resistant to cytotoxic anticancer agents. Herein, we describe a case of treatment of anticancer drug-resistant MSI-high locally advanced colon cancer and review cases of complete response to immune checkpoint inhibitor therapy for colorectal cancer.

**Case presentation:**

A 57-year-old woman was referred to our hospital with a large tumor in the descending colon and extensive thoracic and abdominal wall involvement, including the ribs and diaphragm. No distant metastasis was observed. The tumor had perforated the abdominal wall and formed an abscess. Upon visiting our hospital, emergency surgery was performed. An abdominal wall incision was made to drain the abscess and laparoscopic colostomy was performed. Histopathological examination of biopsy specimens revealed an adenocarcinoma with positive immunohistochemical expressions of both CDX2 and CK20. The patient was diagnosed with a descending colon cancer. Genetic examination found MSI-high, Kras mutation (F12G), and wild-type BRAF. After the inflammation improved, chemotherapy with the FOLFIRI regimen was initiated, but the tumor grew rapidly. As a second-line treatment, nivolumab and ipilimumab combination therapy was initiated. After four cycles of these therapies, the patient was administered nivolumab alone for five cycles. Tumor shrinkage was observed and radical surgery was performed. The patient underwent laparoscopic descending colon and partial thoracic and abdominal wall resection. The abdominal wall muscle was dissected from the abdominal cavity, and subcutaneous tissues, diaphragm, ribs were dissected from the body surface. Pathological examination revealed mucus components, fibrous tissues, and no malignant cells, indicating a complete pathological response (pCR). The patient had a good postoperative course and returned to work after being discharged. No recurrence was observed six months postoperatively.

**Conclusions:**

Herein, we report a case of anticancer drug-resistant MSI-high colon cancer that was resected after treatment with immune checkpoint inhibitors, and a pCR was achieved. This new treatment strategy can be used for the treatment of cases that are not responsive to conventional therapies.

## Background

The standard of care for locally advanced colon cancer is resection of the primary tumor and postoperative adjuvant chemotherapy. The indication for preoperative chemotherapy for resectable colon cancer is unclear, and preoperative chemotherapy for patients with locally advanced colon cancer has been investigated in clinical trials [1]. In the FOxTROT trial, preoperative chemotherapy for patients with T3–4 colon cancer was associated with good postoperative outcomes, but did not lead to a significant difference in survival. More advanced cases, such as T3–4N2–3, may be candidates for preoperative chemotherapy. Preoperative treatment may also be used for locally advanced colon cancer (cT4b) with extensive tumor proximity to the skeletal muscles, major organs, and blood vessels, and tumors that are suspected to invade these organs. Oxaliplatin-based regimens are often used in preoperative chemotherapy. However, microsatellite instability (MSI)-high colorectal cancer is often resistant to cytotoxic anticancer agents. Recently, immune checkpoint inhibitors have been used clinically to treat MSI-high colorectal cancer [2–4], and precision medicine has been practiced.

We report a case of MSI-high locally advanced descending colon cancer with abdominal wall perforation, that was treated with a cytotoxic anticancer drug but rapidly progressed due to anticancer drug resistance. Subsequently, the patient underwent laparoscopic descending colon resection after the commencement of nivolumab and ipilimumab combination therapy.

## Case presentation

**Fig. 1:**
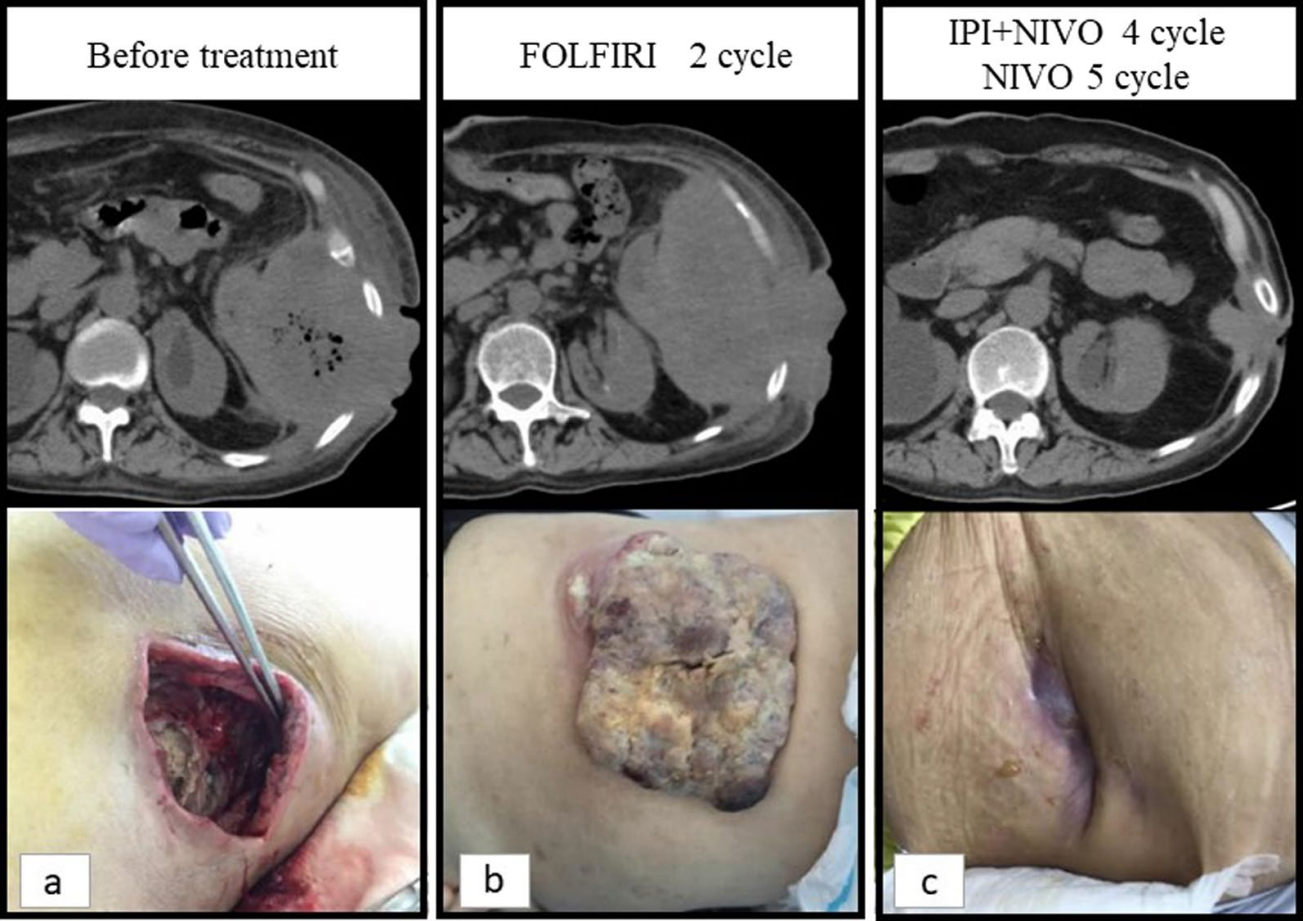
Computed tomography and pictures of the tumor according to the course of anticancer therapy. **a** Before treatment. **b** After two cycles of FOLFIRI therapy. **c** After four cycles of nivolumab and ipilimumab combination therapy and five cycles of nivolumab monotherapy

**Fig. 2:**
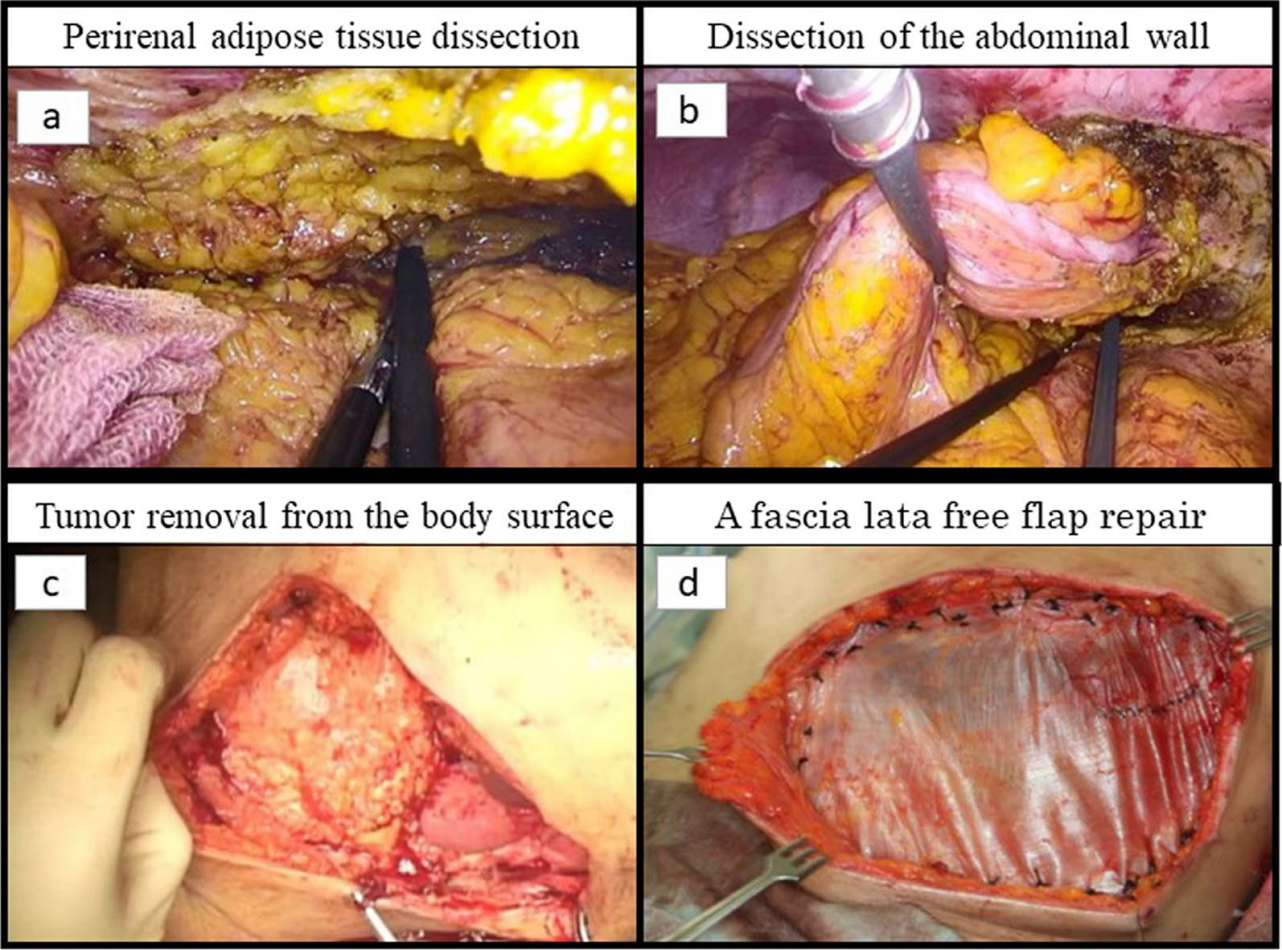
Laparoscopic tumor resection and surgical repair of the thoracic and abdominal wall defect using a fascia lata-free flap. **a** Perirenal adipose tissue dissection. **b** Dissection of the abdominal wall. **c** Tumor removal from the body surface. **d** A fascia lata-free flap repair

**Fig. 3:**
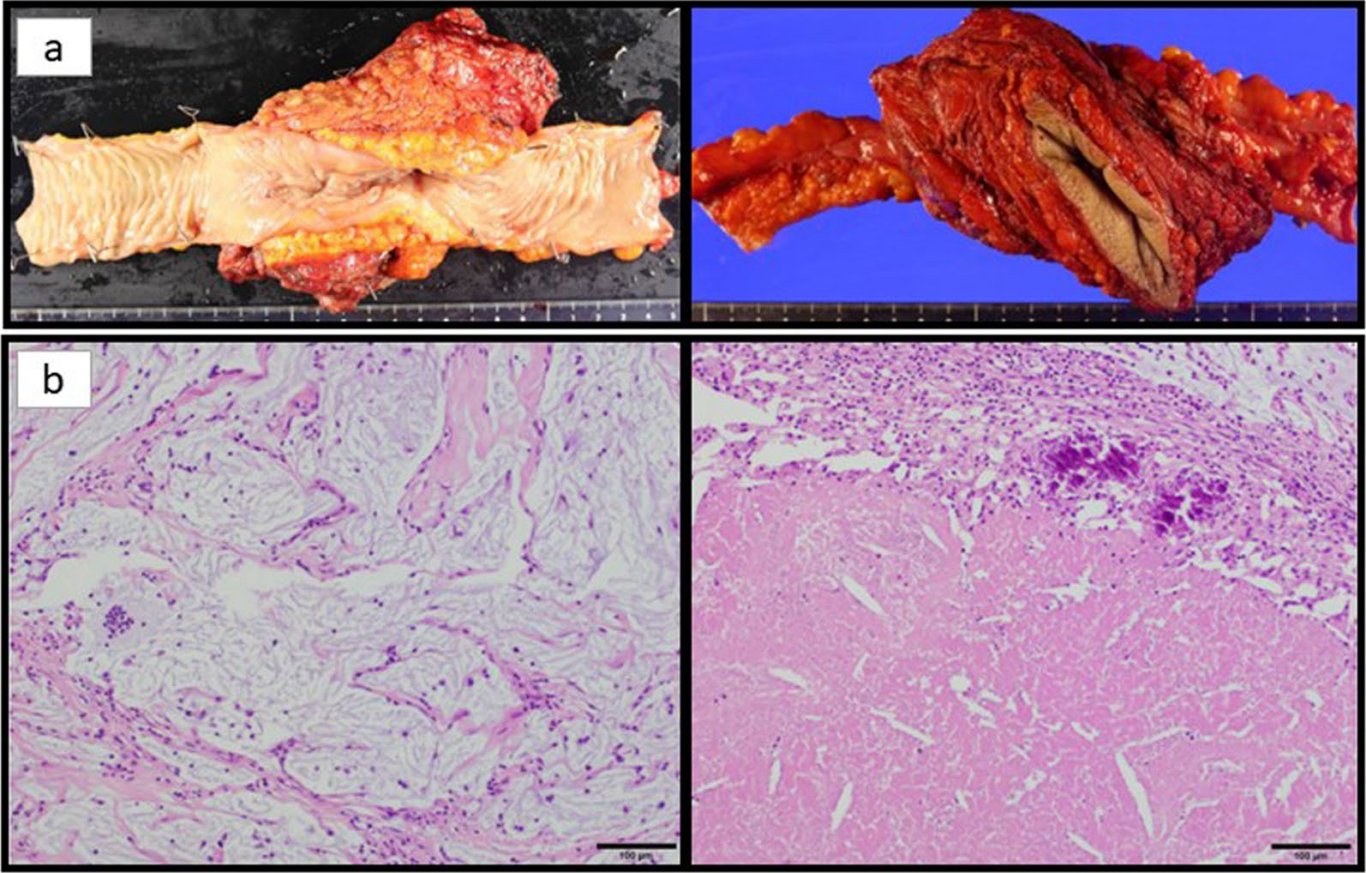
**a** Resected specimen. **b** Pathological findings. Mucus components and fibrous tissue were found in the area where the tumor was thought to have existed, but no malignant cells were found

A 57-year-old woman consulted a physician with a chief complaint of abdominal pain and a history of gallstones, renal pelvic stones, and chronic renal failure. An 18F-fluorodeoxyglucose-positron emission tomography revealed a large tumor in the descending colon with extensive thoracoabdominal wall involvement including the ribs and diaphragm with no distant metastases. The patient was treated with antibacterial agents, but the inflammation did not improve. She was subsequently referred to our hospital. The tumor had perforated the abdominal wall and formed an abscess; therefore, emergency surgery was performed. An abdominal wall incision was made to drain the abscess, and laparoscopic colostomy of the transverse colon was performed. Histopathological examination of biopsy specimens from the tumor revealed well-differentiated and moderately differentiated tubular adenocarcinomas. In addition to primary colon cancer, disseminated lesions from primary pancreatic cancer, gynecology-related adenocarcinoma, and peritoneal cancer were considered as differential diagnoses. The positive expression of both CDX2 and CK20 was confirmed using immunohistochemistry. The patient was subsequently diagnosed with descending colon cancer, cT4b (diaphragm, ribs, abdominal wall muscles, and Gerota’s fascia) N1 M0 cStage IIIc. Genetic examination revealed MSI-high, Kras mutation (F12G), and wild-type BRAF. After the inflammation had resolved, chemotherapy was started to shrink the tumor (Fig. 1a). The creatinine clearance rate on admission was 19.5 mL/min. Due to renal dysfunction, an irinotecan-based regimen (FOLFIRI: irinotecan and 5FU were reduced in one step) instead of an oxaliplatin-based one was selected. After chemotherapy, the creatinine clearance remained at approximately 30 mL/min and did not interfere with the continuation of chemotherapy. However, the tumor grew rapidly after FOLFIRI therapy (Fig. 1b). As a second-line treatment, nivolumab (240 mg) and ipilimumab (1 mg/kg) combination therapy was started. This combination therapy was administered once every 3 weeks. After four cycles of combined therapy, the patient was administered nivolumab (240 mg) alone, according to the optimal clinical use guidelines based on the results of clinical trials. (Fig. 1c). Sufficient tumor shrinkage was observed, and new lesions were not detected after five cycles of nivolumab monotherapy (ycT4b [diaphragm, ribs, abdominal wall muscles, Gerota’s fascia] N0M0 ycStage IIc). An abdominal wall defect associated with tumor resection was deemed tolerable; therefore, radical surgery was performed. After closing the colostomy, the intraperitoneal cavity was laparoscopically observed. Disseminated or metastatic lesions were not observed in the abdominal cavity. The omental bursa was resected, and we confirmed that the tumor had not invaded the spleen or the pancreas. The mesentery of the transverse colon was isolated from the inferior border of the pancreas. The mesocolon and retroperitoneum were dissected from the dorsal side of the inferior mesenteric vein. The left colonic artery, descending mesentery, and descending colon were then dissected. We planned to resect part of the abdominal wall, diaphragm, ribs, and Gerota’s fascia, which had been invaded by the tumor in preoperative treatment, along with the primary tumor. The extent of tumor invasion was confirmed by tissue firmness under laparoscopy, and the abdominal wall was dissected outside the tumor-infiltrated tissue. The dorsal side of the tumor was resected along with perirenal adipose tissues (Fig. 2a). The transversus abdominis muscle was dissected from the abdominal cavity into the external oblique muscle (Fig. 2b). The surgical margins were examined by a pathologist and confirmed to be free from malignant cells intraoperatively. The extent of dissection was confirmed using a needle and was marked on the skin. The skin and ribs were incised, including the fistula, and the abdominal subcutaneous tissue, thoracic wall, and ribs were resected in conjunction with the tumor (Fig. 2c). Intestinal anastomosis was then performed. Preoperative treatment reduced the size of the tumor, thus reducing the extent of resection and the abdominal wall defect. The abdominal wall defect was repaired using a fascia lata-free flap (Fig. 2d) and direct suturing of the skin and diaphragm was performed. Pathological examination revealed mucus components and fibrous tissue in the area where the tumor was thought to have existed, but no malignant cells were found, indicating that a pathological complete response (pCR) had been achieved (Fig. 3). The patient was discharged on postoperative day 14. The patient had a good postoperative course and returned to work after being discharged. Postoperative adjuvant chemotherapy was not administered. No recurrence was observed 6 months postoperatively.

## Discussion

Treatment of advanced colorectal cancer that is resistant to cytotoxic anticancer drugs, including oxaliplatin and irinotecan, and molecular-targeted therapy drugs, is difficult. We successfully treated refractory advanced colorectal cancer using immune checkpoint inhibitors. Our patient had a descending colon cancer with an abscess and extensive thoracoabdominal wall invasion. If surgery had been performed at the time of diagnosis, the patient would have had extensive thoracic and abdominal wall defects, and her postoperative quality of life might have been poor. However, preoperative chemotherapy was administered to reduce the tumor size. In Japan, BRAF, RAS, and MSI levels are commonly measured before chemotherapy, and 5FU combined with irinotecan or oxaliplatin is often used. In addition, anti-EGFR antibodies are used for RAS wild-type, and angiogenesis inhibitors are used for RAS mutants. Oxaliplatin is contraindicated in patients with chronic renal failure (creatinine clearance < 30 mL/min). Currently, immune checkpoint inhibitors have been shown to be safe and effective as first-line therapy for MSI-high colorectal cancer and immune checkpoint inhibitors may be a treatment option. However, immune checkpoint inhibitors were not approved as first-line therapy, when the anticancer therapy was initiated in this case. Oxaliplatin was not used because of the presence of renal dysfunction, and anti-vascular endothelial growth factor antibody was not used because of the need for wound healing in this case. Chemotherapy with the FOLFIRI regimen was initiated, but the tumor grew rapidly; thus, immune checkpoint inhibitors were used as alternatives. In a clinical trial, check Mate 142, the response rates to nivolumab alone and nivolumab plus ipilimumab were 31% and 55%, respectively [3]. In this case, nivolumab and ipilimumab combination therapy was used because tumor shrinkage was desired, although adverse events have been shown to be more frequent with combination therapies.

Laparoscopic surgery was performed and extensive invasion of the abdominal wall was dissected with a good field of view. This technique was particularly advantageous for resecting the area from the cranial to the dorsal side of the tumor, which was considered difficult to view because of the tumor’s location.

MSI-high colorectal cancer corresponds to CMS1 in the CMS classification, and is considered to be poorly responsive to anticancer therapy [5]. MSI-high cancers possess between 10- and 100-fold the mutation load compared with microsatellite stable (MSS) cancers, and MSI-high cancers are highly infiltrated with T cells relative to MSS cancers. This type of immune microenvironment enhances the therapeutic effect of programmed cell death 1 (PD-1) inhibitors [6]. The results of a clinical trial on treatment with an anti-PD-1 antibody (dostarlimab) for MSI-high Stage I–III lower rectal cancer have been reported. Twelve patients were treated with anti-PD-1 antibody monotherapy and all achieved complete clinical remission [7]. In addition to the 12 cases in that study, a literature search using PubMed revealed that in 14 cases, the tumors were resected after preoperative administration of immune checkpoint inhibitors for MSI-high colorectal cancer, and 10 resected specimens and four biopsies showed pCR. The mean age of the 10 patients was 36.2 years. Five patients were male and five were female. The tumor locations in these patients were the left-sided colon in eight cases and the right-sided colon in two patients. Three patients with left-sided colon cancer were treated with preoperative chemotherapy and the mean tumor diameter was 93 mm. Pathological differentiation was reported in eight patients: four well or moderately differentiated tubular adenocarcinomas and four poorly differentiated or mucinous carcinomas. Five patients survived, and were reported without recurrence. Two of the four patients in whom pCR was confirmed in biopsy specimens were being managed for rectal cancer, and were followed-up without surgery [8–17] (Table 1).

**Table 1:** Summary of case reports of pathologic complete response after treatment with immune checkpoint inhibitors for colorectal cancer

Case	Age	Sex	Location	BRAF	RAS	Patho	Diameter	Drug	Cycle	Line	Pathological examination	Survival	Characteristic	References
Biopsy cases														
1	54	M	C	Mut	Wt	N.A	41	10 mg/kg pembro	16	3	Distant LN	> 2y	BRAF mutant CRC	Discov Med. 2016 21(117):341–7
2	81	M	R	–	–	Por	53	Pembro	11	NAC	Rectum	17 m		J Natl Compr Canc Netw. 2020 18(7):798–804. [8]
3	55	M	A	–	–	N.A	30	IPI Nivo	3	NAC	Rectum	12 m	TMB 21.3 mut./megabase	J Natl Compr Canc Netw. 2020 18(7):798–805. [8]
4	62	W	R			N.A	N.A	Nivo	6	2	Rectum	1y	Lynch syndrome	Oncoimmunology. 2019 19;8(12):e1663109. [13]
Resection cases														
1	38	F	R	–	–	Tub2/Muc	N.A	FOLFOX + pembro	7	NAC	Rectum	10 m	Lynch syndrome Combination therapy	J Natl Compr Canc Netw. 2020 18(7):798–806. [8]
2	50	M	A	Wt	Wt	Tub2	90	Nivo	18 m	2	Peritoneal recurrence	9 m	Cytoreductive surg for peritoneal recurrence	BMC Gastroenterol. 2022 10;22(1):17 [9]
3	28	F	R	Wt	Mut	N.A	N.A	Nivo	12	2	Rectum	N.A	Dense lymphoplasmacytic infiltrate	J Cancer Res Ther 2021;17(6):1552–1555. [10]
4	33	M	R	–	–	Tub2	N.A	IPI Nivo	1	NAC	Rectum	N.A	Lynch syndrome, Stage III MSH2 (-) and MSH6 (-)	Oncologist.2021;26(12):e2110-e2114. [11]
5	27	F	R	–	–	Tub1	N.A	IPI Nivo	3	1	Rectum	6 m	RT Lynch syndrome,	Eur J Cancer. 2020;135:75–77. [12]
6	27	M	R	Wt	Wt	Muc	80	Nivo	6	NAC	Rectum	> 1y	TMB high, ATR, PMRM1, ARID2, SMARCA4 mut	Oncoimmunology. 2019 19;8(12):e1663108. [13]
7	45	M	S	–	–	Tub2	73	FOLFOX + pembro	6	3	Sigmoid colon	N.A		Oncology. 2022 8;36(2):115–119. [14]
8	45	M	A	–	–	Por	110	Pembro	20	2	Peritoneal meta	14 m	Peritoneal metastasis	Clin J Gastroenterol. 2022;15(1):134–139. [15]
9	41	F	S	Wt	Mut	N.A	113	Pembro	12	3	Liver	N.A	Lynch syndrome,	Chemotherapy. 2018 Apr 5;63(2):90–94. [16]
10	28	F	S	Wt	Mut	Por	N.A	FOLFOX + pembro	2 m	1	Colon, liver, LN	N.A	FoundationOne	Clin Colorectal Cancer. 2018 Jun;17(2):e229-e232. [17]

*mut* mutant, *wt* wild type, *N.A.* not available, *pembro* pembrolizumab, *LN* lymph node, *NAC* neoadjuvant chemotherapy

Although MSI-high colon cancer accounts for only 4–15% of cases, it is often resistant to anticancer agents and is difficult to treat. We performed laparoscopic resection for MSI-high descending colon cancer after treatment with immune checkpoint inhibitors. The surgery was safely performed laparoscopically, and the postoperative course was good. Future clinical trials on perioperative treatment of MSI-high colon cancer using immune checkpoint inhibitors are warranted.

## Conclusions

We reported a case of MSI-high locally advanced descending colon cancer with abdominal wall perforation that was treated with cytotoxic anticancer drugs but had rapidly progressed due to anticancer drug resistance. Subsequently, the patient underwent laparoscopic descending colon resection after the administration of nivolumab and ipilimumab combination therapy, and pCR was achieved.

## Data Availability

Not applicable.

## Abbreviations

MSI: Microsatellite instability
pCR: Pathological complete response
CMS: Consensus molecular subtype
MSS: Microsatellite stable
